# ﻿Three new *Stenohya* species with sexually dimorphic leg I from China (Pseudoscorpiones, Neobisiidae)

**DOI:** 10.3897/zookeys.1204.123294

**Published:** 2024-06-04

**Authors:** Jiaqi Zhao, Xiangbo Guo, Feng Zhang

**Affiliations:** 1 Key Laboratory of Zoological Systematics and Application, College of Life Sciences, Hebei University, Baoding, Hebei 071002, China Hebei University Baoding China; 2 Hebei Basic Science Center for Biotic Interaction, Hebei University, Baoding, Hebei 071002, China Hebei University Baoding China

**Keywords:** Diversity, fused podomeres, sexual dimorphism, taxonomy

## Abstract

Three new species of the genus *Stenohya* Beier, 1967 from China are described: *Stenohyagibba***sp. nov.** and *S.papillata***sp. nov.** from Hunan Province, and *S.guangmingensis***sp. nov.** from Jiangxi Province. In addition to their sexually dimorphic pedipalp, these three new species also have a uniquely sexual dimorphic leg I, which has not been reported in other *Stenohya* species. Additionally, an updated key to the Chinese *Stenohya* species is provided.

## ﻿Introduction

*Stenohya* Beier, 1967, originally placed in the family Hyidae (Beier 1967), was erected with the type species *S.vietnamensis* Beier, 1967 and transferred to the family Neobisiidae by Harvey (1991), based on the presence of venom apparatus only in the fixed chelal finger and the presence of a non-lanceolate trichobothrium *t.* Of the 23 *Stenohya* species known, 14 of them occur in China (WPC 2022; Li and Shi 2023). These 14 species mainly occur in the southern region of China, except for *S.xiningensis*, which is lives in the northern region. *Stenohya* mainly lives in leaf litter and soil, under rocks, bark, and fern fronds.

Sexual dimorphism is common in *Stenohya* species and is mainly reflected in the morphology of the pedipalps. Male pedipalps are distinctly thinner than the female ones in *S.huangi* Hu & Zhang, 2012, *S.martensi* (Schawaller, 1987), and *S.pengae* Hu & Zhang, 2012 (Schawaller 1987; Hu and Zhang 2012; Zhan et al. 2023). The shapes of pedipalpal femora differ in males and females in *S.arcuata* Guo, Zang & Zhang, 2019 and *S.tengchongensis* Yang & Zhang, 2013 (Yang and Zhang 2013; Guo et al. 2019). Male padipalpal chelal hands have special prominences near the base of the fingers in *S.bicornuta* Guo, Zang & Zhang, 2019, *S.curvata* Zhao, Zhang & Jia, 2011, *S.hamata* (Leclerc & Mahnert, 1988), and *S.meiacantha* Yang & Zhang, 2013 (Leclerc and Mahnert 1988; Zhao et al. 2011; Yang and Zhang 2013; Guo et al. 2019). The males of *S.spinata* Zhan, Feng & Zhang, 2023 have spinous apophyses on the dorsal side of the median pedipalpal chelal hand, and strong thorns on the femur and patella but these are absent in the females (Zhan et al. 2023).

In this study, three new *Stenohya* species with sexually dimorphic pedipalps and leg I are described from China: *S.gibba* sp. nov., *S.papillata* sp. nov., and *S.guangmingensis* sp. nov.

## ﻿Materials and methods

All specimens were preserved in 75% alcohol. Temporary slide mounts were prepared in glycerol. Detailed examinations were carried out with an Olympus BX53 general optical microscope. Photographs and measurements were taken using a Leica M205A stereomicroscope equipped with a Leica DFC550 camera. Drawings were made using the Inkscape ver. 1.0.2.0. Figures were edited and formatted using Adobe Photoshop 2022. The specimens were deposited in the Museum of Hebei University (**MHBU**), Baoding, China.

Terminology and measurements largely follow Chamberlin (1931), except for the nomenclature of the pedipalps and legs, and the terminology of trichobothria (Harvey 1992); the term “rallum” (for flagellum) is adopted from Judson (2007). The following abbreviations are used in the text for the trichobothria: ***b*** = basal; ***sb*** = sub-basal; ***st*** = sub-terminal; ***t*** = terminal; ***ib*** = interior basal; ***isb*** = interior sub-basal; ***ist*** = interior sub-terminal; ***it*** = interior terminal; ***eb*** = exterior basal; ***esb*** = exterior sub-basal; ***est*** = exterior sub-terminal; ***et*** = exterior terminal.

## ﻿Taxonomy

**Family Neobisiidae Chamberlin, 1930**

**Subfamily Microcreagrinae Balzan, 1892**

**Genus *Stenohya* Beier, 1967**

### ﻿ Stenohya gibba sp. nov.

Taxon classificationAnimaliaPseudoscorpionesNeobisiidae

21A1F709-3666-5834-B062-4EBA78DCD559

https://zoobank.org/96FB4752-3A00-430C-9F00-7EC80CA53888

**Figure 1.**
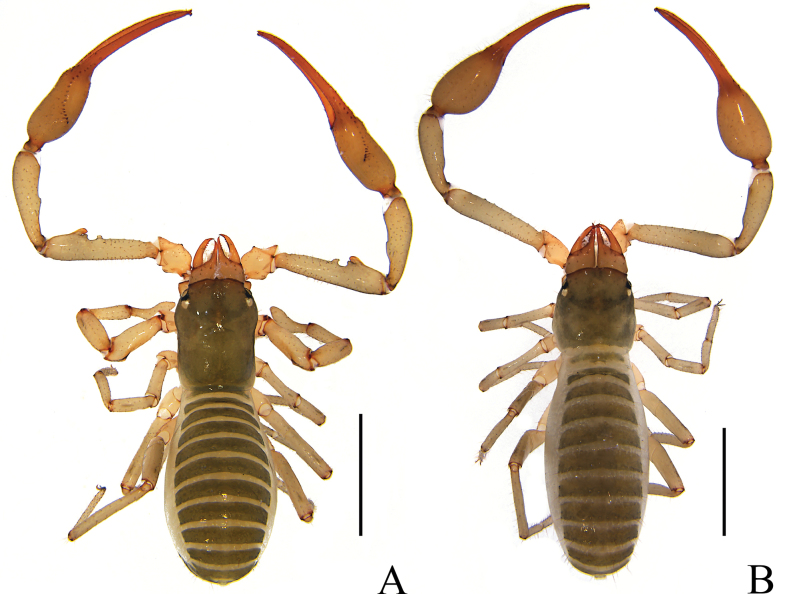
*Stenohyagibba* sp. nov. **A** holotype male (dorsal view) **B** paratype female (dorsal view). Scale bars: 2 mm.

**Figure 2.**
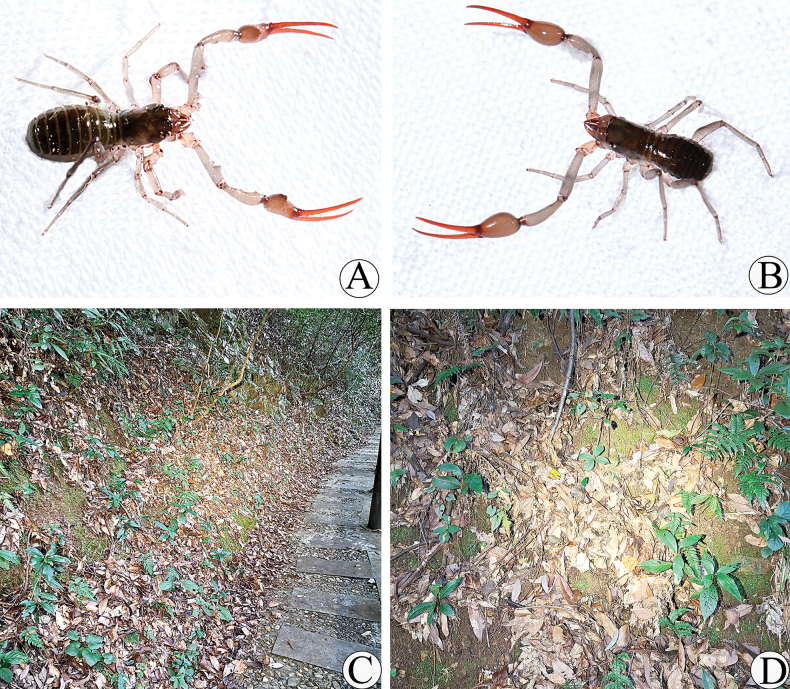
Type locality and habitus of *Stenohyagibba* sp. nov. **A** male habitus **B** female habitus **C, D** litter layer inhabited by habitus.

**Figure 3.**
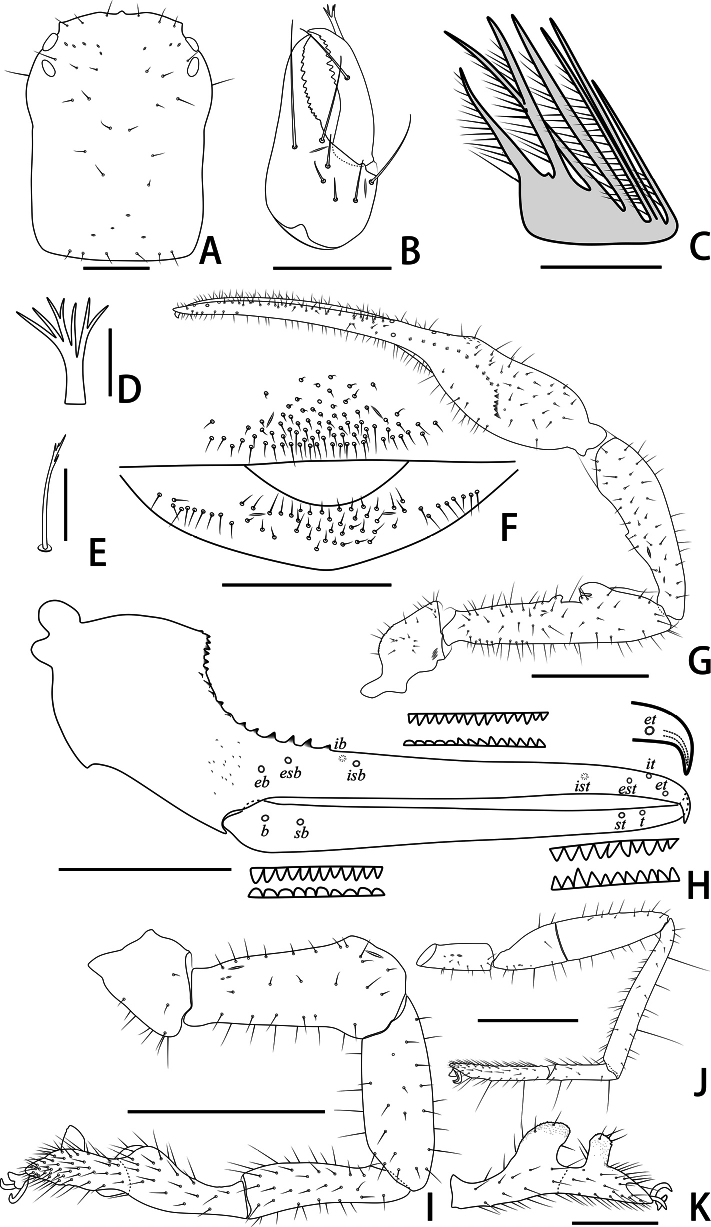
Holotype male of *Stenohyagibba* sp. nov. **A** carapace, dorsal view **B** right chelicera, dorsal view **C** rallum **D** galea **E** subterminal tarsal seta **F** chaetotaxy of genital area, ventral view **G** right pedipalp, dorsal view **H** right chela, lateral view, showing trichobothriotaxy, teeth and venom apparatus **I** right leg I, lateral view **J** right leg IV, lateral view **K** right leg I (basitarsus and telotarsus), retrolateral view. Scale bars: 0.5 mm (**A, B, F, K**); 0.1 mm (**C–E**); 1 mm (**G–J**).

**Figure 4.**
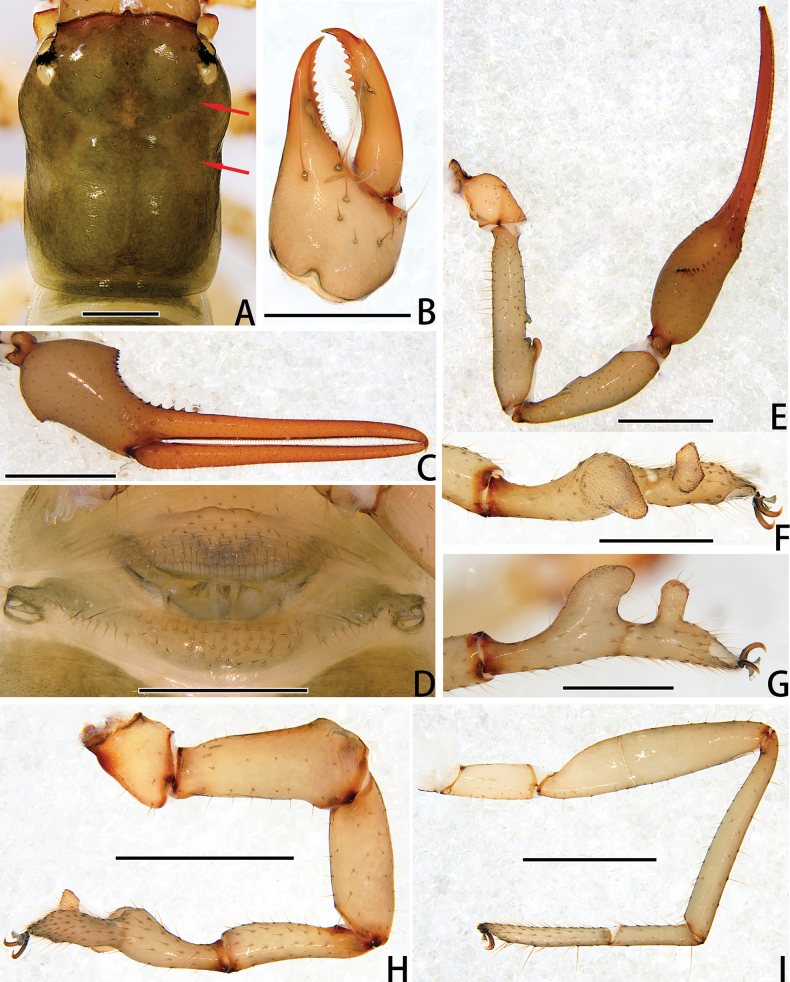
Holotype male of *Stenohyagibba* sp. nov. **A** carapace, dorsal view (red arrows showing two transverse grooves) **B** right chelicera, dorsal view **C** right chela, lateral view **D** genital area, ventral view **E** right pedipalp, dorsal view **F** right leg I (basitarsus and telotarsus), prolateral view **G** right leg I (basitarsus and telotarsus), retrolateral view. **H** right leg I, lateral view **I** right leg IV, lateral view. Scale bars: 0.5 mm (**A, B, D, F, G**); 1 mm (**C, E, H, I**).

**Figure 5.**
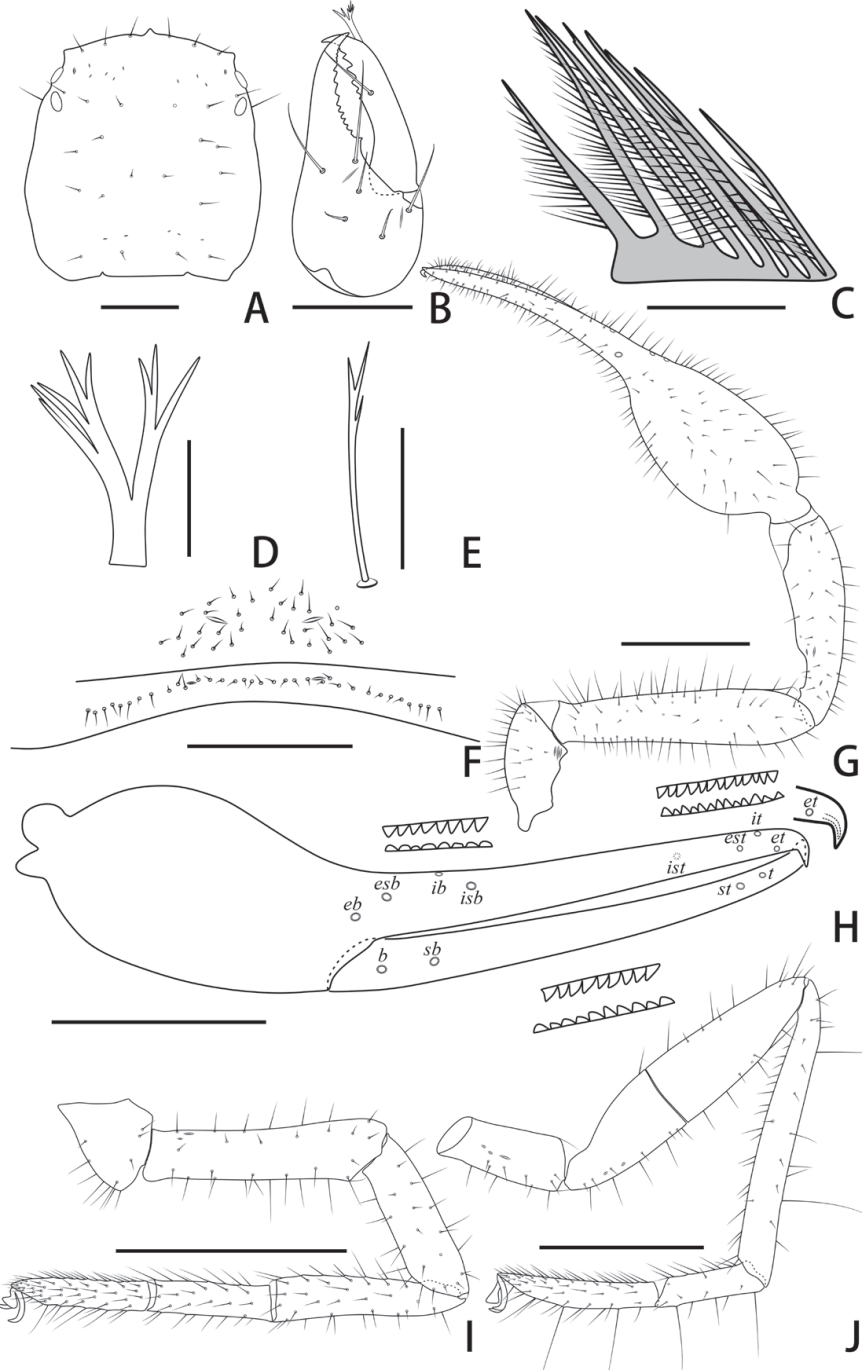
Paratype female of *Stenohyagibba* sp. nov. **A** carapace, dorsal view **B** right chelicera, dorsal view **C** rallum **D** galea **E** subterminal tarsal seta **F** chaetotaxy of genital area, ventral view **G** right pedipalp, dorsal view **H** right chela, lateral view, showing trichobothriotaxy, teeth and venom apparatus **I** right leg I, lateral view **J** right leg IV, lateral view. Scale bars: 0.5 mm (**A, B, F**); 0.1 mm (**C–E**); 1 mm (**G–J**).

**Figure 6.**
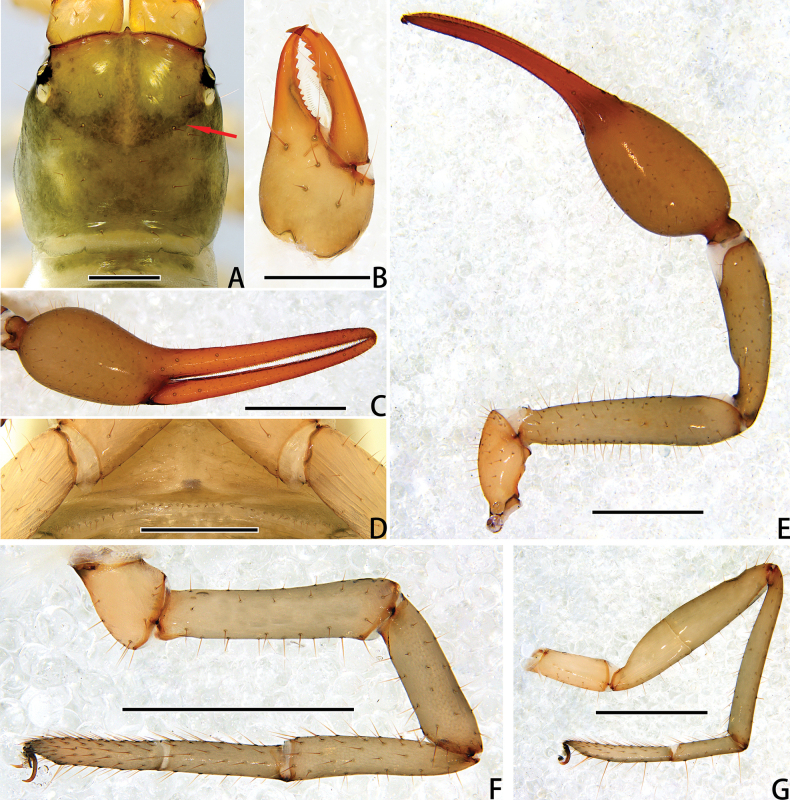
Paratype female of *Stenohyagibba* sp. nov. **A** carapace, dorsal view (red arrow showing transverse groove) **B** right chelicera, dorsal view **C** right chela, lateral view **D** genital area, ventral view **E** right pedipalp, dorsal view **F** right leg I, lateral view **G** right leg IV, lateral view. Scale bars: 0.5 mm (**A, B, D**); 1 mm (**C, E–G**).

Figs 1 , 2 , 3 , 4 , 5 , 6 Chinese name. 驼峰狭伪蝎

#### Type material.

***Holotype*** male (Ps.-MHBU-HN2023111901), China: Hunan Province, Suining County, Huangsang Nature Reserve in Nanshan National Park [26°24'32"N, 110°05'38"E], 460 m a.s.l., 19 November 2023, in leaf litter (Fig. 2C, D), Jiaqi Zhao, Jianzhou Sun, Tao Zheng & Songtao Shi leg. ***Paratypes***: three males (Ps.-MHBU-HN2023111902–04), four females (Ps.-MHBU-HN2023013105–08), same data as for holotype.

#### Etymology.

The specific name is derived from the Latin word “*gibbus*”, meaning hump-shaped, which refers to the shape of the projections on the basitarsus and telotarsus of the male leg I.

#### Diagnosis.

Carapace with four well-developed eyes, epistome triangular (Figs 3A, 4A, 5A, 6A). Male pedipalpal trochanter with a process and small frosted projections on the prolateral position (Figs 3G, 4E); femur with three projections (Figs 3G, 4E); patella with a small projection medially on the prolateral position (Figs 3G, 4E); chelal hand concaved distally at the dorsal side, with 15–18 small, triangular apophyses on the dorsal side, extending into the dorsal face of fixed finger (Figs 3H, 4C). Male leg I specialized, femur and patella enlarged, basitarsus and telotarsus semi-fused, the dividing line between the two limb segments visible, basitarsus and telotarsus each with a large columnar projection laterally (Figs 3I, K, 4F–H). Female pedipalpal movable chelal finger with 79–87 teeth; female pedipalpal chela (with pedicel) 4.67–4.98 times longer than wide.

#### Description.

**Adult male** (holotype and male paratypes) (Figs 1A, 2A).

***Carapace*** (Figs 3A, 4A). Carapace 1.30–1.36 times longer than broad, with a total of 30–32 setae, including six near anterior margin and 6–7 near posterior margin; eight lyrifissures near the eyes, four lyrifissures near posterior margin; epistome small, triangular, with rounded top; with four corneate eyes. Carapace divided into three parts by two transverse, shallow grooves, the anterior part uplifted, the median part smooth, the posterior part uplifted, and with microgrooves.

***Chelicera*** (Figs 3B, 4B). Hand with 6–7 setae and two lyrifissures, movable finger with one seta; fixed finger with 13–15 teeth; movable finger with 7–9 teeth; serrula exterior with 44–46 lamellae; serrula interior with 36–37 lamellae; galea developed, divided into two main branches, one branch five, while the other two (Fig. 3D); rallum consisting of 7–8 blades, all with anteriorly directed spinules, the basal-most blade shortest (Fig. 3C).

***Pedipalps*** (Figs 3G, H, 4C, E). Apex of pedipalpal coxa rounded, with seven long setae. Trochanter with a process on the median prolateral position, as well as some small frosted projections; femur with a curved cylindrical process on the median prolateral position, as well as a projection on the subdistal prolateral surface, and with a columnar process adjacent to this projection; patella with a small projection on the median prolateral position and two lyrifissures (Figs 3G, 4E); chelal hand deeply concaved at the dorsal side of distal half, with 15–18 small, triangular, spinous apophyses arranged in a row on the dorsal side, each spinous apophysis with a seta at the base, a few spinous apophyses extended into the dorsal face of fixed finger. On the posterior side, several small granular processes located at the distal of the hand and near the base of the fixed finger, at the ventral of the hand from the distal to two-thirds with shallow invagination. Fixed chelal finger slightly curved upward at median to distal part (Figs 3H, 4C). Trochanter 1.46–1.65, femur 3.96–4.37, patella 3.47–3.71, chela with pedicel 3.98–4.16, chela without pedicel 3.71–3.89 times longer than broad, movable finger 1.98–2.35 times longer than hand without pedicel. Fixed chelal finger with eight, movable chelal finger with four trichobothria: *eb* and *esb* situated on the base of hand, grouped very closely with *ib* and *isb*; *est*, *et* and *it* grouped distally; *ist* closer to *est*-*et*-*it* than to *isb*-*ib*-*esb*-*eb* in fixed chelal finger; *b* and *sb* situated closer to each other in basal half, *st* and *t* close to each other in distal half of movable finger. Venom apparatus present only in fixed chelal finger, venom duct short. Fixed chelal finger with 117 pointed teeth, movable finger with 103–108 teeth, 47–51 rounded teeth at base, and 56–57 pointed teeth at distal position.

***Abdomen*.** Pleural membrane granulated. Tergites and sternites undivided, tergal chaetotaxy (I–XI): 4–5: 7–8: 7–11: 9–10: 9–10: 10–12: 11–12: 11–12: 11–12: 12: 11, sternal chaetotaxy (IV–XI): 22–28: 21–24: 19–24: 18–19: 19: 17–19: 12–15: 4, sternites VI–VIII with 3–6 medial scattered glandular setae, anal cone with two dorsal and two ventral setae. Genital area (Figs 3F, 4D): anterior genital sternite with 75–80 setae and two lyrifissures; posterior genital sternite with 55–59 setae and two lyrifissures.

***Legs*** (Figs 3I–K, 4F–I). Leg I specialized, femur and patella enlarged, basitarsus and telotarsus semi-fused, the dividing line between the two limb segments visible, basitarsus and telotarsus each with a large columnar projection on the lateral side (Figs 3I, K, 4F–H), femur with three lyrifissures. Leg IV generally typical, long, and sinewy, trochanter with three lyrifissures (Figs 3J, 4I). Leg I: trochanter 1.40–1.55, femur 2.06–2.24, patella 2.61–3.27, tibia 3.25–3.43, basitarsus 2.31–2.52, telotarsus 2.52–2.89 times longer than deep. Leg IV: trochanter 2.23–2.73, femur + patella 4.08–4.72, tibia 6.96–7.52, basitarsus 4.19–4.38, telotarsus 6.67–7.15 times longer than deep; tibia with two submedial tactile setae (TS = 0.60–0.67, 0.96), basitarsus with two tactile setae (TS = 0.12–0.14, 0.82–0.83), telotarsus with two tactile setae (TS = 0.20, 0.55–0.58); subterminal tarsal seta bifurcate (Fig. 3E). Arolium not divided, shorter than the slender and simple claws.

**Adult female** (paratype females) (Figs 1B, 2B): mostly same as males, except where noted.

***Carapace*** (Figs 5A, 6A). Carapace 1.02–1.19 times longer than broad, with a total of 29–30 setae, including six near anterior margin and 4–5 near posterior margin; ten lyrifissures near the eyes, five lyrifissures near posterior margin; the front half of carapace uplifted, the back half smooth and with triangular invagination at 1/3 and 2/3 positions.

***Chelicera*** (Figs 5B, 6B). Fixed finger with 14–15 teeth; movable finger with 6–7 teeth; serrula exterior with 45–51 lamellae; serrula interior with 38–40 lamellae; galea divided into two main branches, one branch five, while the other three (Fig. 5D); rallum consisting of 8–9 blades, all with anteriorly directed spinules, the basal-most blade shortest (Fig. 5C).

***Pedipalps*** (Figs 5G, H, 6C, E). Apex of pedipalpal coxa with six long setae. Femur with a few tubercles prolaterally. Trochanter 1.87–1.94, femur 4.56–4.80, patella 3.15–3.71, chela with pedicel 4.67–4.98, chela without pedicel 3.94–4.14 times longer than broad, movable finger 1.60–1.72 times longer than hand without pedicel. Fixed chelal finger with 99–100 pointed teeth, movable finger with 79–87 teeth, 39–44 rounded teeth at base, and 40–43 pointed ones.

***Abdomen*.** Tergal chaetotaxy (I–XI): 3–5: 7–8: 8–10: 9–10: 11: 10–11: 10–13: 11–12: 11–12: 11–13: 8–10, sternal chaetotaxy (IV–XI): 22–24: 21–24: 18–20: 18–20: 16–18: 16–18: 13–14: 4–6, sternites VI–VIII with two medial scattered glandular setae; genital area (Figs 5F, 6D): sternite II with total of 29–34 setae and two lyrifissures; sternite III with a row of 31–34 setae and two lyrifissures along posterior margin.

***Legs*** (Figs 5I, J, 6F, G). Leg I: trochanter 1.25–1.37, femur 3.64–3.96, patella 3.00–3.45, tibia 3.79–4.56, basitarsus 3.29–4.00, telotarsus 4.77–4.85 times longer than deep. Leg IV: trochanter 2.55–2.79, femur + patella 4.30–4.72, tibia 6.68–7.29, basitarsus 3.93–4.40, telotarsus 6.53–6.79 times longer than deep; tibia with two submedial tactile setae (TS = 0.22–0.29, 0.72–0.76), basitarsus with two tactile setae (TS = 0.12–0.13, 0.80), telotarsus with two tactile setae (TS = 0.20–0.22, 0.59).

#### Measurements

**(in mm; length/breadth or, for legs, length/depth). Male** (holotype and paratypes). Body length 4.55–5.30. Carapace 1.84–1.90/1.40–1.42. Pedipalpal trochanter 0.76–0.86/0.48–0.57, femur 1.82–2.01/0.46, patella 1.56–1.67/0.45–0.46, chela with pedicel 3.80–3.87/0.92–0.99, chela without pedicel 3.58–3.67/0.92–0.99, hand without pedicel length 1.15–1.30, movable finger length 2.58–2.70. Leg I: trochanter 0.56–0.59/0.38–0.40, femur 1.03–1.14/0.50–0.52, patella 0.92–1.08/0.33–0.36, tibia 0.78–0.83/0.23–0.25, basitarsus 0.53–0.60/0.21–0.26, telotarsus 0.55–0.58/0.19–0.23. Leg IV: trochanter 0.69–0.81/0.26–0.31, femur + patella 1.63–1.84/0.39–0.40, tibia 1.58–1.64/0.21–0.23, basitarsus 0.64–0.70/0.15–0.16, telotarsus 0.93–1.00/0.13–0.15.

**Female** (paratypes). Body length 4.45–5.65. Carapace 1.48–1.64/1.24–1.55. Pedipalpal trochanter 0.83–0.91/0.43–0.47, femur 1.96–2.06/0.41–0.44, patella 1.45–1.66/0.41–0.48, chela with pedicel 3.56–3.72/0.84–0.90, chela without pedicel 3.38–3.55/1.39–1.52, hand without pedicel length 1.27–1.37, movable finger length 2.17–2.30. Leg I: trochanter 0.41–0.46/0.30–0.36, femur 0.95–1.02/0.24–0.28, patella 0.66–0.78/0.22–0.24, tibia 0.72–0.82/0.18–0.19, basitarsus 0.46–0.60/0.14–0.15, telotarsus 0.58–0.63/0.12–0.13. Leg IV: trochanter 0.74–0.87/0.29–0.34, femur + patella 1.74–1.85/0.38–0.43, tibia 1.47–1.56/0.21–0.22, basitarsus 0.59–0.70/0.15–0.16, telotarsus 0.95–0.98/0.14–0.15.

#### Distribution.

China (Hunan).

#### Remarks.

The male of this new species differs from all other species of the genus *Stenohya* by the presence of a large columnar projection on the lateral side of basitarsus and telotarsus. The female can be distinguished from other *Stenohya* species reported from China by the presence of 79–87 teeth on pedipalpal movable chelal finger (115–118 in *S. arcuatа*; 68 in *S.bomica*; 96–98 in *S.hainanensis*; 46–51 in *S.huangi*; 45–55 in *S.pengae*), the pedipalpal chela with pedicel 4.67–4.98 times longer than wide (4.20 in *S.bicornuta*; 4.19–4.37 in *S.curvata*; 4.16–4.27 in *S.hainanensis*; 3.56 in *S.meiacantha*; 4.09–4.25 in *S.pengae*; 4.02–4.10 in *S.spinata*; 3.44–4.50 in *S.tengchongensis*) (Zhao and Zhang 2011; Zhao et al. 2011; Hu and Zhang 2012; Yang and Zhang 2013; Guo and Zhang 2016; Guo et al. 2019; Zhan et al. 2023).

### ﻿ Stenohya papillata sp. nov.

Taxon classificationAnimaliaPseudoscorpionesNeobisiidae

0D772C1F-E2EA-5D82-8DDD-74EBD4F39AC9

https://zoobank.org/50230EC7-A562-4638-AA98-DA00F4B0F59A

**Figure 7.**
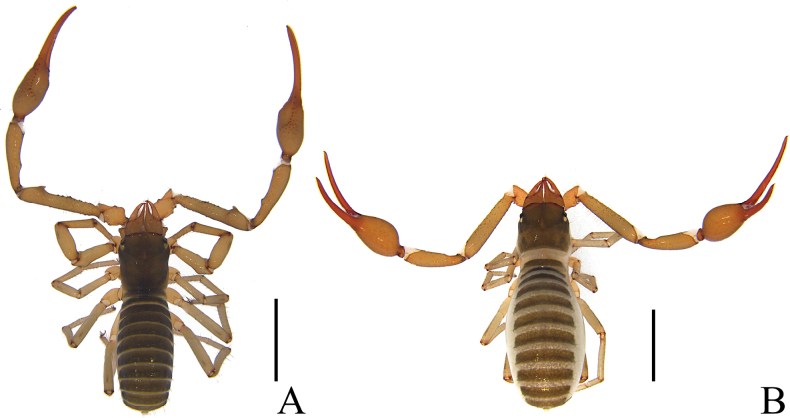
*Stenohyapapillata* sp. nov. **A** holotype male (dorsal view) **B** paratype female (dorsal view). Scale bars: 2 mm.

**Figure 8.**
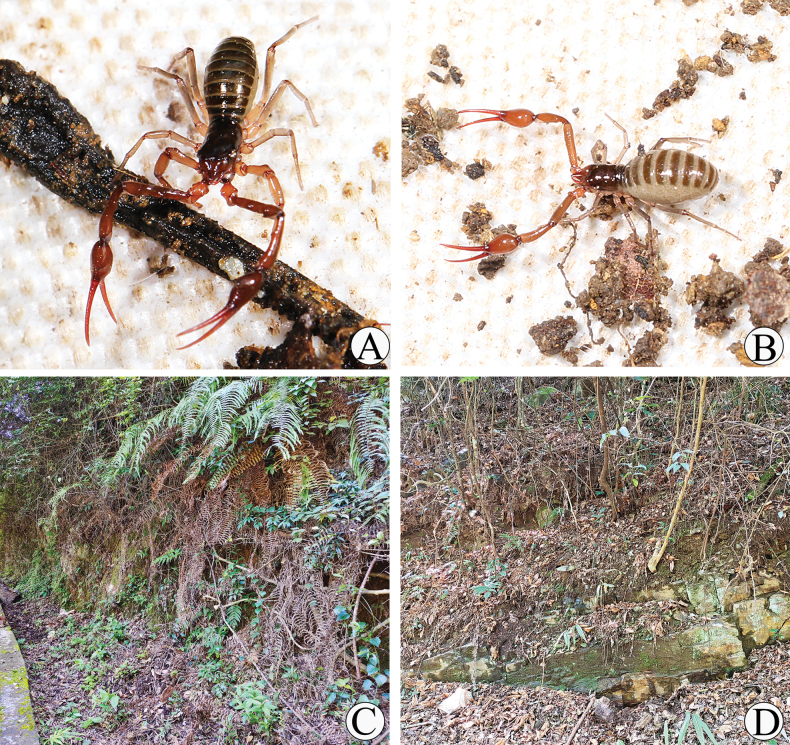
Type locality and habitus of *Stenohyapapillata* sp. nov. **A** male habitus **B** female habitus **C, D** litter layer inhabited by habitus.

**Figure 9.**
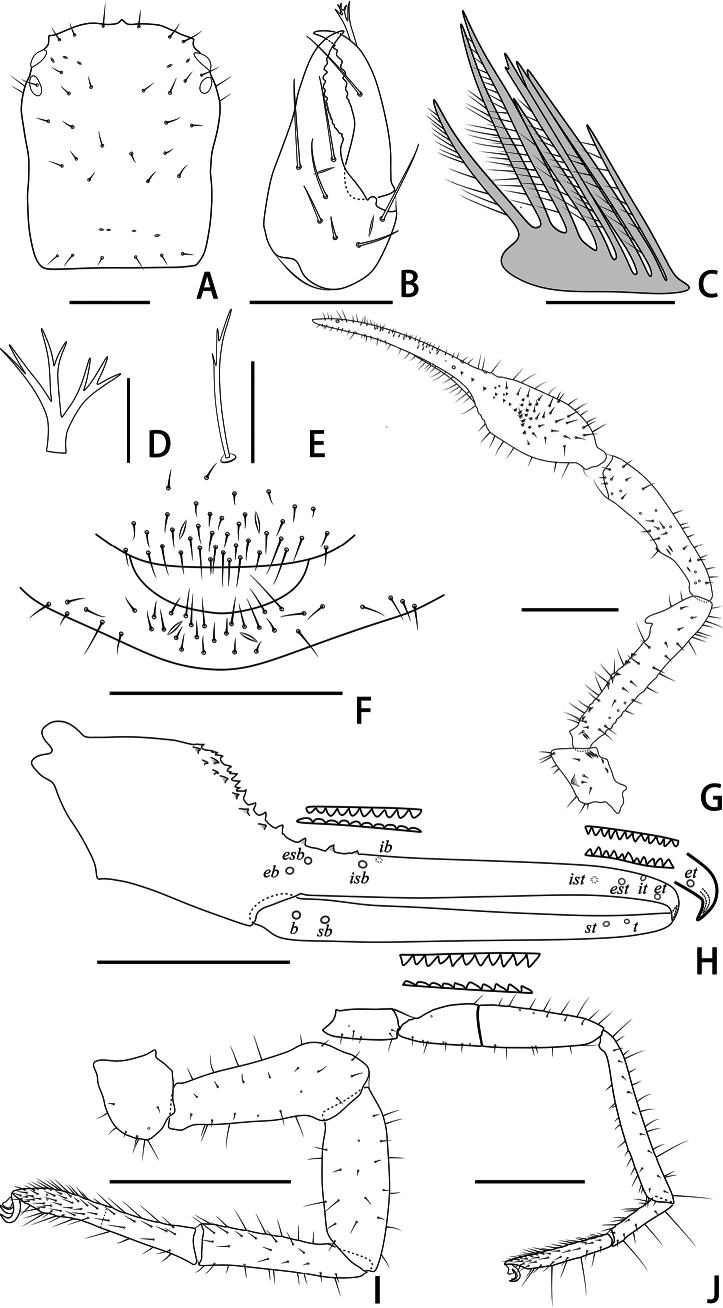
Holotype male of *Stenohyapapillata* sp. nov. **A** carapace, dorsal view **B** right chelicera, dorsal view **C** rallum **D** galea **E** subterminal tarsal seta **F** chaetotaxy of genital area, ventral view **G** right pedipalp, dorsal view **H** right chela, lateral view, showing trichobothriotaxy, teeth and venom apparatus **I** right leg I, lateral view **J** right leg IV, lateral view. Scale bars: 0.5 mm (**A, B, F**); 0.1 mm (**C–E**); 1 mm (**G–J**).

**Figure 10.**
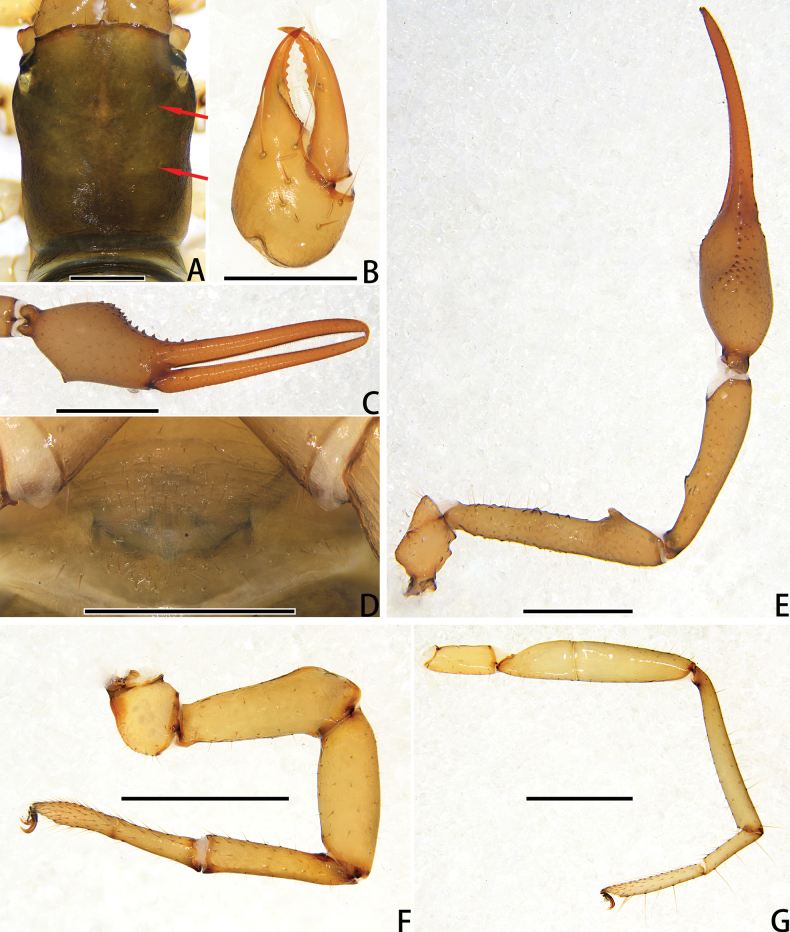
Holotype male of *Stenohyapapillata* sp. nov. **A** carapace, dorsal view (red arrows showing two transverse grooves) **B** right chelicera, dorsal view **C** right chela, lateral view **D** genital area, ventral view **E** right pedipalp, dorsal view **F** right leg I, lateral view **G** right leg IV, dorsal view. Scale bars: 0.5 mm (**A, B, D**); 1 mm (**C, E–G**).

**Figure 11.**
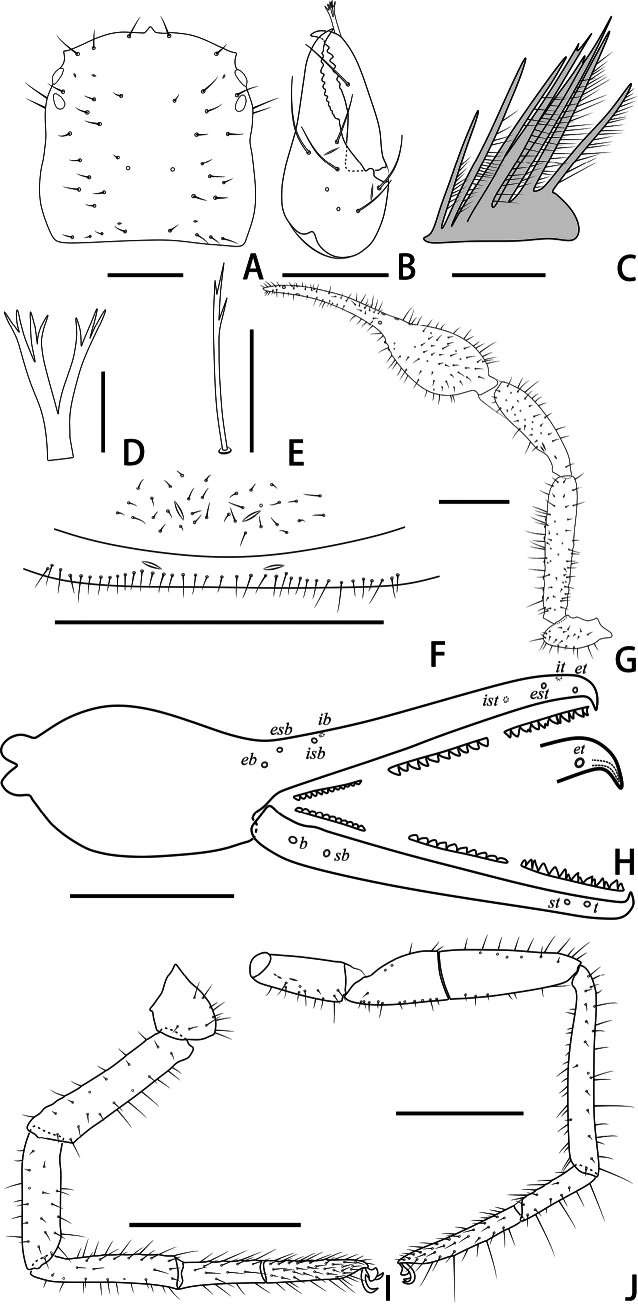
Paratype female of *Stenohyapapillata* sp. nov. **A** carapace, dorsal view **B** right chelicera, dorsal view **C** rallum **D** galea **E** subterminal tarsal seta **F** chaetotaxy of genital area, ventral view **G** right pedipalp, dorsal view **H** right chela, lateral view, showing trichobothriotaxy, teeth and venom apparatus **I** left leg I, lateral view **J** right leg IV, lateral view. Scale bars: 0.5 mm (**A, B**); 0.1 mm (**C–E**); 1 mm (**F–J**).

**Figure 12.**
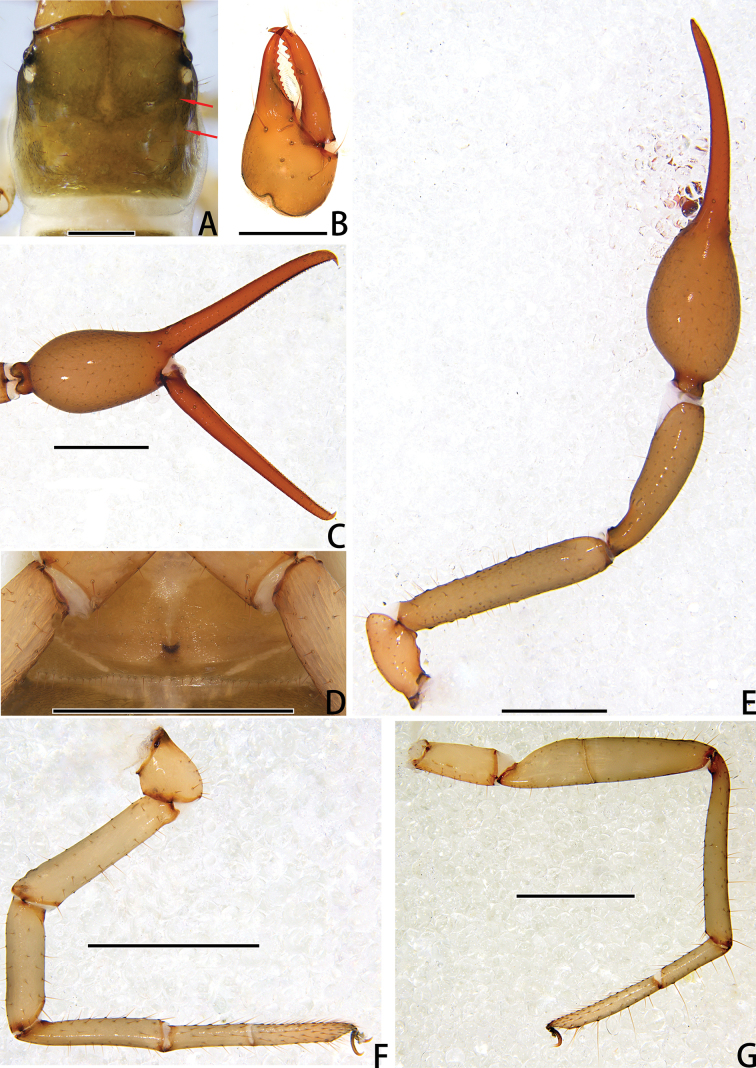
Paratype female of *Stenohyapapillata* sp. nov. **A** carapace, dorsal view (red arrows showing two transverse grooves) **B** right chelicera, dorsal view **C** right chela, lateral view **D** genital area, ventral view **E** right pedipalp, dorsal view **F** left leg I, lateral view **G** right leg IV, lateral view. Scale bars: 0.5 mm (**A, B**); 1 mm (**C–G**).

Figs 7 , 8 , 9 , 10 , 11 , 12 Chinese name. 乳突狭伪蝎

#### Type material.

***Holotype*** male (Ps.-MHBU-HN2023111909), China: Hunan Province, Suining County, Ganchong Village [26°29'59"N, 110°08'01"E], 460 m a.s.l., 19 November 2023, in leaf litter (Fig. 8C, D), Jiaqi Zhao, Jianzhou Sun, Tao Zheng & Songtao Shi leg. ***Paratypes***: two males (Ps.-MHBU-HN2023111910–11), three females (Ps.-MHBU-HN2023111912–14), same data as for holotype.

#### Etymology.

The specific name is derived from the Latin word “*papillatus*” and refers to the presence of a papillary projection on the ventral face of the pedipalpal chela hand in male.

#### Diagnosis.

Carapace with four well-developed eyes, epistome triangular (Figs 9A, 10A, 11A, 12A). Male pedipalpal trochanter with a small process and small frosted projections on the median prolateral position; femur with several big tubercles and a projection on the prolateral position, a few small tubercles at the retrolateral surface; patella with a triangular protuberance on the prolateral position (Figs 9G, 10E); chelal hand concaved distally at the dorsal side, with 30–33 triangular spinous apophyses on the dorsal side and a papillary projection at the median of ventral side (Figs 9H, 10C). Male leg I femur and patella enlarged and basitarsus and telotarsus semi-fused (Figs 9I, 10F). Female pedipalpal fixed chelal finger with 99–102 teeth; pedipalpal femur 4.76–4.98 times longer than wide.

#### Description.

**Adult male** (holotype and male paratypes) (Figs 7A, 8A).

***Carapace*** (Figs 9A, 10A). Carapace 1.23–1.36 times longer than broad, with a total of 36–37 setae, including six near anterior margin and 6–7 near posterior margin; five lyrifissures near the eyes, four lyrifissures near posterior margin; epistome small, triangular, with rounded apex; four well-developed eyes; carapace divided into three parts by two transverse, shallow grooves, the anterior part uplifted, the median part with microgrooves, the posterior part uplifted and with microgrooves.

***Chelicera*** (Figs 9B, 10B). Hand with 6–7 setae and two lyrifissures, movable finger with one seta; fixed finger with 16–17 teeth; movable finger with six teeth; serrula exterior with 48–55 lamellae; serrula interior with 39–42 lamellae; galea developed, one branch four, while the other three (Fig. 9D); rallum consisting of eight blades, all with anteriorly directed spinules, the basal-most blade shortest (Fig. 9C).

***Pedipalps*** (Figs 9G, H, 10C, E). Apex of pedipalpal coxa rounded, with six long setae. Trochanter with a small process on the median prolateral position, as well as some small frosted projections; femur with several big tubercles on the prolateral position, as well as a projection on the subdistal prolateral surface, few small tubercles placed at the retrolateral surface; patella with a projection on the prolateral position and three lyrifissures (Figs 9G, 10E); chelal hand concaved at the dorsal side of distal half, and with 30–33 triangular-shaped, spinous apophyses on the dorsal side, every apophyse with a setae at the base (Figs 9H, 10C). A few spinous apophyses extended to the subbase of fixed finger. A papillary projection in the middle of the ventral aspect of the pedipalpal chelal hand. On the posterior side, a few small granular processes dispersedly located at the distal of the hand and near the base of the fingers. Fixed chelal finger slightly curved upward at median to distal part (Figs 9H, 10C). Trochanter 1.46–1.63, femur 4.48–5.02, patella 3.67–4.78, chela with pedicel 4.58–4.60, chela without pedicel 3.53–3.71 times longer than broad, movable finger 1.68–1.84 times longer than hand without pedicel. Fixed chelal finger with eight, movable chelal finger with four trichobothria: *eb* and *esb* situated on the base of hand, grouped very closely with *ib* and *isb*; *est*, *et* and *it* grouped distally; *ist* closer to *est*-*et*-*ist* than to *isb*-*ib*-*esb*-*eb* in fixed chelal finger; *b* and *sb* situated closer to each other in basal half, *st* and *t* close to each other in distal half of movable finger. Venom apparatus present only in fixed chelal finger, venom duct short. Fixed chelal finger with 93–105 pointed teeth, movable finger with 87–94 teeth, 34–42 rounded teeth at base, and 52–53 pointed ones.

***Abdomen*.** Pleural membrane granulated. Tergites and sternites undivided, tergal chaetotaxy (I–XI): 5: 7: 9–10: 10–11: 10–11: 11: 11: 11–12: 12: 11–12: 12–13, sternal chaetotaxy (IV–XI): 26–30: 19–24: 17: 15–18: 16–18: 14–16: 11–14: 5, sternites VI–VIII with 11 medial scattered glandular setae, anal cone with two dorsal and two ventral setae. Genital area (Figs 9F, 10D): anterior genital sternite with 39–40 setae and two lyrifissures; posterior genital sternite with 31–37 setae and two lyrifissures.

***Legs*** (Figs 9I, J, 10F, G). Leg I specialized, femur and patella enlarged, basitarsus and telotarsus semi-fused, the dividing line between the two limb segments visible. Leg IV generally typical, long, and sinewy. Leg I: trochanter 1.16–1.71, femur 2.56–3.28, patella 2.61–4.10, tibia 4.24–4.75, basitarsus 3.06–3.87, telotarsus 3.29–3.64 times longer than deep. Leg IV: trochanter 2.60–2.81, femur + patella 3.77–5.18, tibia 6.58–7.45, basitarsus 4.20–4.57, telotarsus 5.88–7.07 times longer than deep; tibia with two submedial tactile setae (TS = 0.68–0.74, 0.96–1.03), basitarsus with two tactile setae (TS = 0.09–0.11, 0.53–0.56), telotarsus with two tactile setae (TS = 0.14–0.17, 0.84–0.88); subterminal tarsal seta bifurcate (Fig. 9E). Arolium not divided, shorter than the slender and simple claws.

**Adult female** (paratype females) (Figs 7B, 8B): mostly same as males, except where noted.

***Carapace*** (Figs 11A, 12A). Carapace 1.04–1.13 times longer than broad, with a total of 39–42 setae, including 5–6 near anterior margin and 8–9 near posterior margin; three lyrifissures near the eyes, two lyrifissures near posterior margin; carapace divided into three parts by two transverse, shallow grooves, the anterior part uplifted, the median part smooth, the posterior part uplifted, and with microgrooves.

***Chelicera*** (Figs 11B, 12B). Fixed finger with 12–15 teeth; movable finger with 6–7 teeth; serrula exterior with 49–51 lamellae; serrula interior with 36–44 lamellae.

***Pedipalps*** (Figs 11G, H, 12C, E). Apex of pedipalpal coxa with eight long setae. Femur with several tubercles on the prolateral position, as well as a few small tubercles placed lateral surface. Trochanter 1.89–2.09, femur 4.76–4.98, patella 3.10–3.55, chela with pedicel 3.89–4.27, chela without pedicel 3.86–4.09 times longer than broad, movable finger 1.56–1.72 times longer than hand without pedicel. Fixed chelal finger with 99–102 pointed teeth, movable finger with 90–94 teeth, 43–49 rounded teeth at base, and 45–47 pointed ones.

***Abdomen*.** Tergal chaetotaxy (I–XI): 6: 7–8: 10–11: 12: 11: 11–13: 12: 11–12: 11–12: 12–13: 12, sternal chaetotaxy (IV–XI): 22–24: 20–22: 18–19: 17–19: 17–19: 15–18: 12: 4–5, sternites VI–VIII with 2–3 medial scattered glandular setae; genital area (Figs 11F, 12D): sternite II with total of 27–35 setae and two lyrifissures; sternite III with a row of 35–38 setae and two lyrifissures along posterior margin.

***Legs*** (Figs 11I, J, 12F, G). Leg I: trochanter 1.18–1.74, femur 3.06–4.35, patella 2.96–3.57, tibia 4.61–5.31, basitarsus 3.57–4.33, telotarsus 4.14–4.69 times longer than deep. Leg IV: trochanter 3.03–3.24, femur + patella 3.93–5.05, tibia 5.88–7.71, basitarsus 4.13–5.00, telotarsus 5.88–9.00 times longer than deep; tibia with three submedial tactile setae (TS = 0.20–0.30, 0.69–0.77, 0.98–1.06), basitarsus with two tactile setae (TS = 0.14–0.15, 0.83–0.88), telotarsus with two tactile setae (TS = 0.25–0.32, 0.57–0.61).

#### Measurements

**(in mm; length/breadth or, for legs, length/depth)**. **Male** (holotype and paratypes). Body length 4.00–4.53. Carapace 1.50–1.70/1.16–1.28. Pedipalpal trochanter 0.76–0.80/0.49–0.52, femur 2.02–2.06/0.41–0.46, patella 1.69–1.81/0.36–0.46, chela with pedicel 3.24–3.40/0.83–0.92, chela without pedicel 3.08–3.25/0.83–0.92, hand without pedicel length 1.13–1.30, movable finger length 2.08–2.23. Leg I: trochanter 0.50–0.59/0.31–0.43, femur 1.05–1.17/0.32–0.43, patella 0.86–1.09/0.21–0.39, tibia 0.87–0.95/0.20–0.21, basitarsus 0.49–0.58/0.15–0.17, telotarsus 0.46–0.51/0.14. Leg IV: trochanter 0.78–0.87/0.25–0.31, femur + patella 1.74–1.86/0.34–0.47, tibia 1.47–1.64/0.21–0.24, basitarsus 0.63–0.73/0.14–0.17, telotarsus 0.94–0.99/0.14–0.16.

**Female** (paratypes). Body length 3.77–6.12. Carapace 1.41–1.56/1.33–1.46. Pedipalpal trochanter 0.83–0.92/0.44–0.47, femur 2.00–2.14/0.42–0.43, patella 1.56–1.71/0.44–0.51, chela with pedicel 3.59–3.70/0.85–0.89, chela without pedicel 3.40–3.48/0.85–0.89, hand without pedicel length 1.36–1.40, movable finger length 2.18–2.34. Leg I: trochanter 0.45–0.49/0.27–0.38, femur 0.95–1.04/0.23–0.31, patella 0.77–0.82/0.23–0.26, tibia 0.81–0.85/0.16–0.18, basitarsus 0.50–0.52/0.12–0.14, telotarsus 0.58–0.61/0.13–0.14. Leg IV: trochanter 0.81–0.91/0.25–0.29, femur + patella 1.81–1.92/0.37–0.46, tibia 1.53–1.62/0.21–0.26, basitarsus 0.65–0.70/0.14–0.16, telotarsus 0.96–1.00/0.11–0.17.

#### Distribution.

China (Hunan).

#### Remarks.

Similar to *S.gibba* in having specialized leg I in male, this new species can be distinguished by the morphology of the pedipalpal chelal hand and leg I. The male of this new species has 30–33 dentate convex on the dorsal side, a papillary protuberance on the ventral side of chelal hand, and lacks the projection on the basitarsus and telotarsus of leg I, but the male *S.gibba* has 15–18 dentate convex, which arranged in a row on the dorsal side of chelal hand and a large columnar projection on the basitarsus and telotarsus in leg I. Female of this new species can be easily distinguished from the other *Stenohya* species in having 99–102 teeth on pedipalpal fixed chelal finger (124–129 in *S. arcuatа*; 76 in *S.bomica*; 85–90 in *S.curvata*; 88–89 in *S.hainanensis*; 63–69 in *S.huangi*; 84 in *S.meiacantha*; 66–79 in *S.pengae*; 82–91 in *S.spinata*; 81–89 in *S.tengchongensis*), and pedipalpal femur 4.76–4.98 times longer than wide (4.23–4.45 in *S. arcuatа*; 5.37 in *S.bicornuta*; 5.00–5.24 in *S.curvata*; 6.07–6.32 in *S.huangi*; 5.13 in *S.meiacantha*; 5.18–5.83 in *S.pengae*; 4.00–4.13 in *S.tengchongensis*), and pedipalpal chela with pedicel 3.89–4.27 times longer than wide (3.50–3.74 in *S. arcuatа*; 4.19–4.37 in *S.curvata*; 4.67–4.98 in *S.gibba*; 3.56 in *S.meiacantha*) (Zhao et al. 2011; Zhao and Zhang 2011; Hu and Zhang 2012; Yang and Zhang 2013; Guo and Zhang 2016; Guo et al. 2019; Zhan et al. 2023).

### ﻿ Stenohya guangmingensis sp. nov.

Taxon classificationAnimaliaPseudoscorpionesNeobisiidae

A75B21E8-E887-58EA-82D3-BBA85CF45E89

https://zoobank.org/61EEA057-CB4E-48D9-A9BF-98FD1D9AE0C4

**Figure 13.**
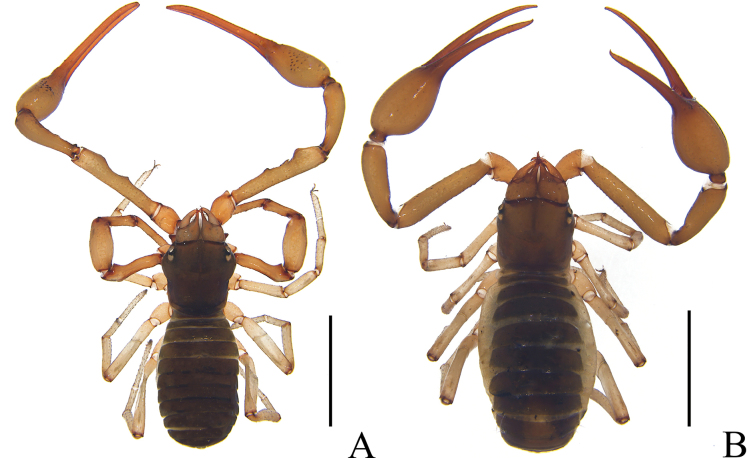
*Stenohyaguangmingensis* sp. nov. **A** holotype male (dorsal view) **B** paratype female (dorsal view). Scale bars: 2 mm.

**Figure 14.**
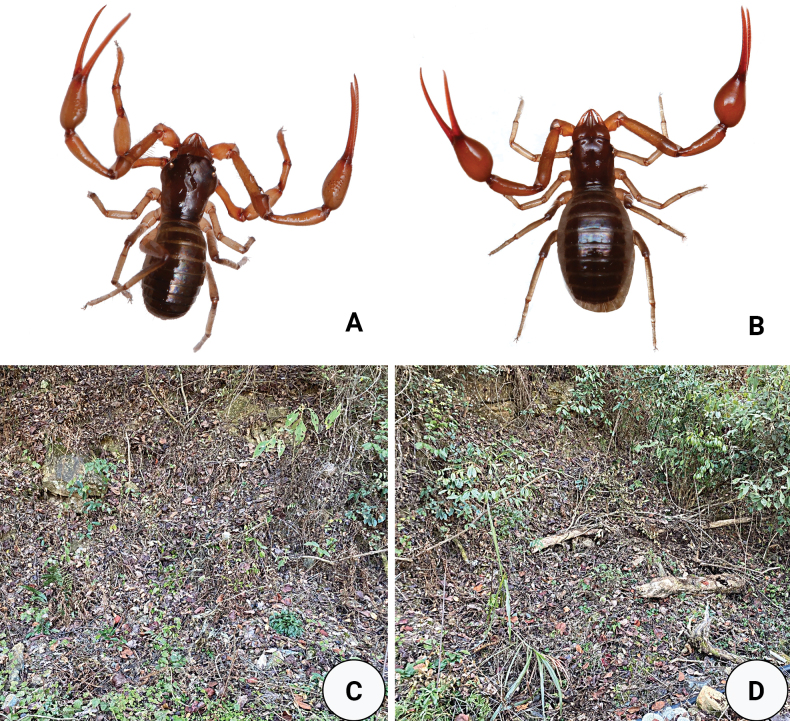
Type locality and habitus of *Stenohyaguangmingensis* sp. nov. **A** male habitus **B** female habitus **C–D** litter layer inhabited by habitus.

**Figure 15.**
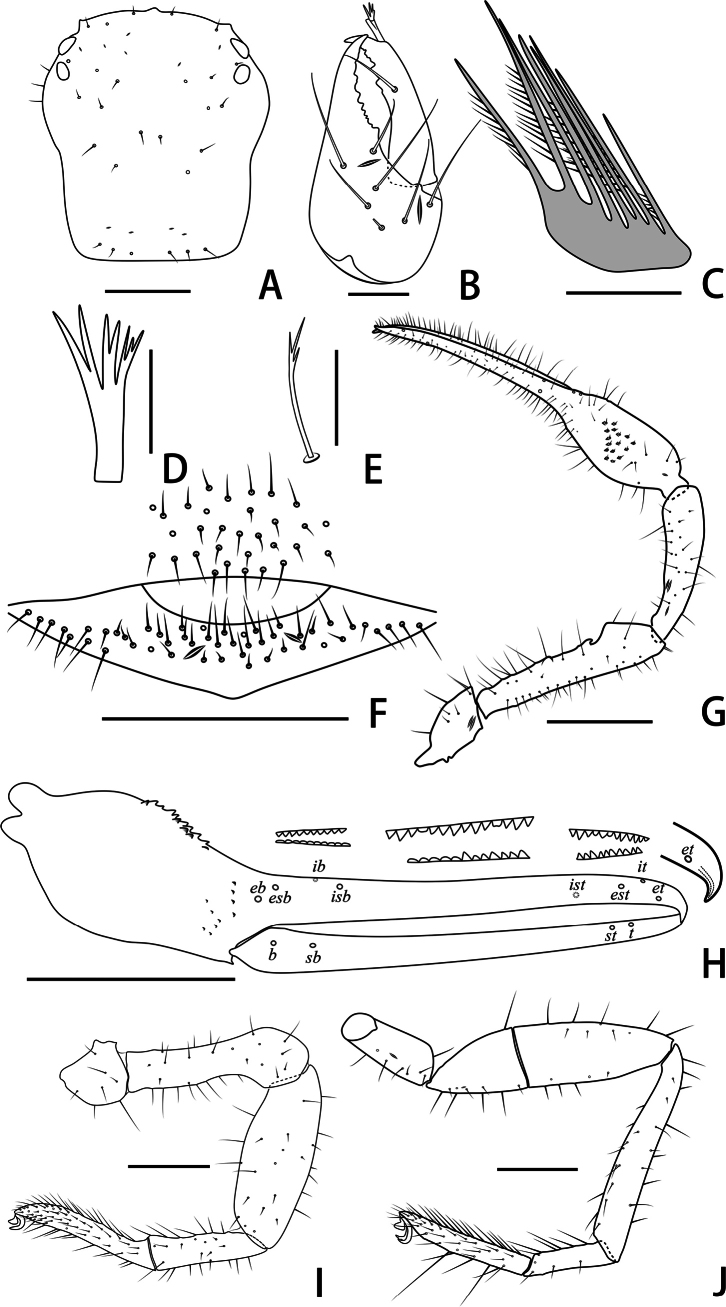
Holotype male of *Stenohyaguangmingensis* sp. nov. **A** carapace, dorsal view **B** right chelicera, dorsal view **C** rallum **D** galea **E** subterminal tarsal seta **F** chaetotaxy of genital area, ventral view **G** right pedipalp, dorsal view **H** right chela, lateral view, showing trichobothriotaxy, teeth and venom apparatus **I** right leg I, lateral view **J** right leg IV, lateral view. Scale bars: 0.5 mm (**A, F, I, J**); 0.2 mm (**B**); 0.1 mm (**C–E**); 1 mm (**G, H**).

**Figure 16.**
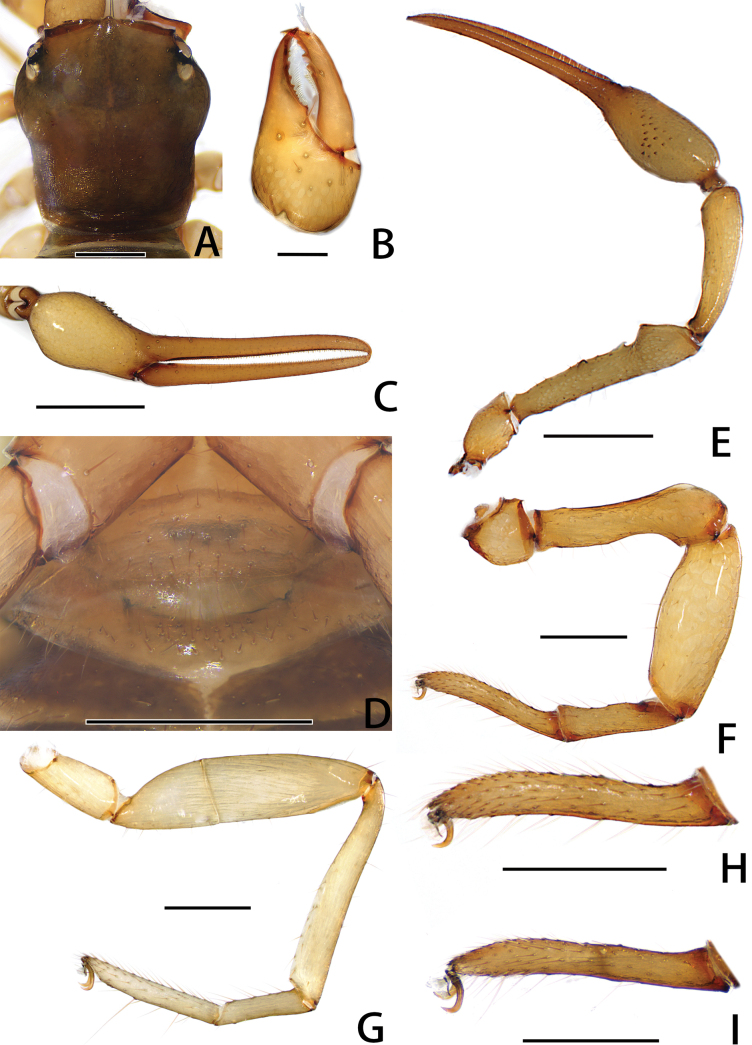
Holotype and paratype male of *Stenohyaguangmingensis* sp. nov. **A–H** holotype male **I** paratype male **A** carapace, dorsal view **B** right chelicera, dorsal view **C** right chela, lateral view **D** genital area, ventral view **E** right pedipalp, dorsal view **F** right leg I, lateral view **G** right leg IV, lateral view **H** right leg I (basitarsus and telotarsus), lateral view **I** right leg I (basitarsus and telotarsus), lateral view (paratype). Scale bars: 0.5 mm (**A, D, F–I**); 0.2 mm (**B**); 1 mm (**C, E**).

**Figure 17.**
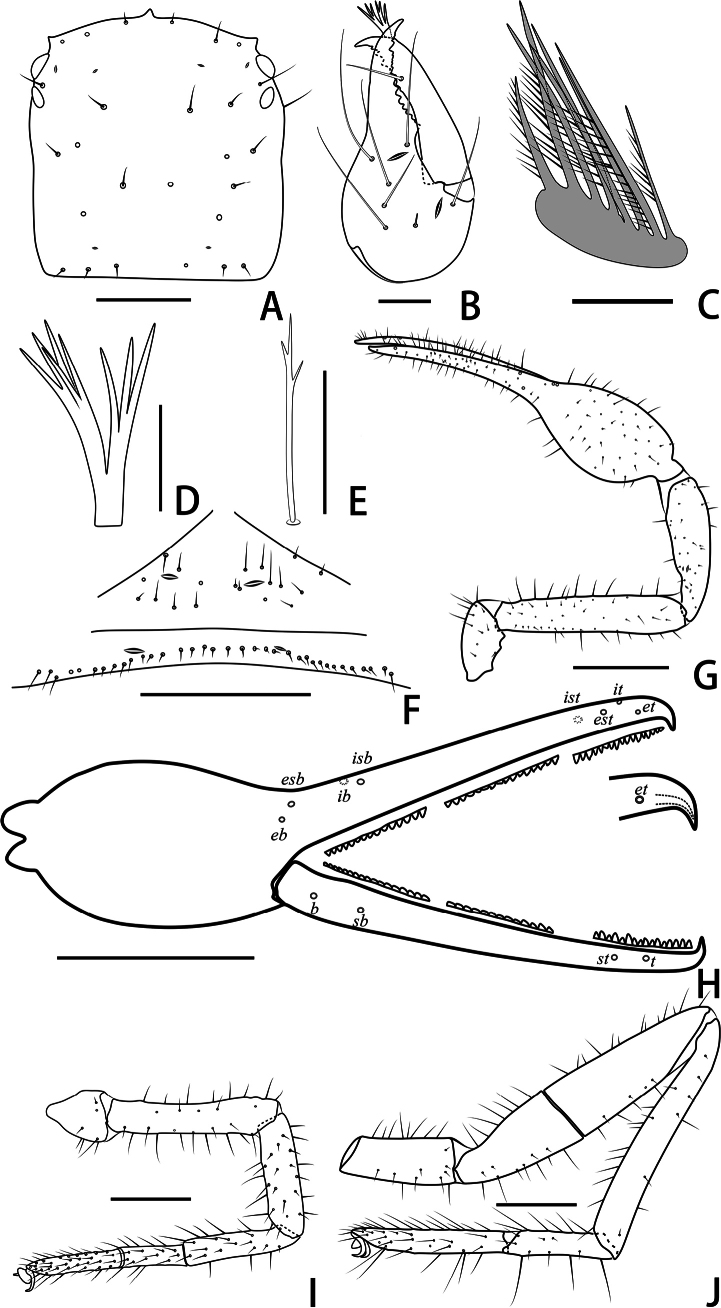
Paratype female of *Stenohyaguangmingensis* sp. nov. **A** carapace, dorsal view **B** right chelicera, dorsal view **C** rallum **D** galea **E** subterminal tarsal seta **F** chaetotaxy of genital area, ventral view **G** right pedipalp, dorsal view **H** right chela, lateral view, showing trichobothriotaxy, teeth and venom apparatus **I** right leg I, lateral view **J** right leg IV, lateral view. Scale bars: 0.5 mm (**A, D, F–H**); 0.2 mm (**B**); 1 mm (**C, E**).

**Figure 18.**
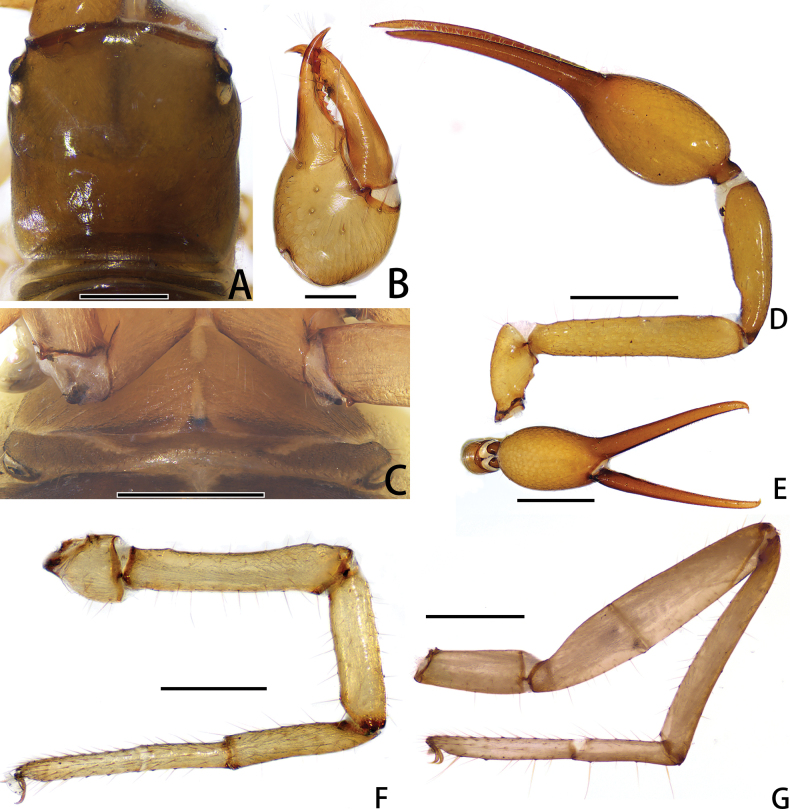
Paratype female of *Stenohyaguangmingensis* sp. nov. **A** carapace, dorsal view **B** right chelicera, dorsal view **C** genital area, ventral view **D** right pedipalp, dorsal view **E** right chela, lateral view **F** right leg I, lateral view **G** right leg IV, lateral view. Scale bars: 0.5 mm (**A, C, F, G**); 0.2 mm (**B**); 1 mm (**D, E**).

Figs 13 , 14 , 15 , 16 , 17 , 18 Chinese name. 光明狭伪蝎

#### Type material.

***Holotype*** male (Ps.-MHBU-JX2023013101), China: Jiangxi Province, Jinggangshan City, Guangming Township, 868 County Road [26°26'04"N, 114°12'11"E], 305 m a.s.l., 31 January 2023, in leaf litter and under rocks (Fig. 14C, D), Xiangbo Guo, Jianzhou Sun, Tao Zheng & Songtao Shi leg. ***Paratypes***: four males (Ps.-MHBU-JX2023013102–05), three females (Ps.-MHBU-JX2023013106–08), same data as for holotype.

#### Etymology.

The specific name refers to the type locality.

#### Diagnosis.

Carapace with four well-developed eyes, epistome triangular (Figs 15A, 16A, 17A, 18A). Male pedipalpal femur with a large tubercle in the median area, two subdistal projections on the prolateral surface; patella smooth; chelal hand with 23 small, triangular, spinous apophyses on the dorsal side (Figs 15G, H, 16C, E). Male leg I femur with an inward depression at the distal part, patella enlarged (Figs 15I, 16F). Female carapace 1.02–1.10 times longer than broad; carapace with a total of 29–30 setae; apex of pedipalpal coxa with six long setae; pedipalpal patella 3.39–3.46; pedipalpal movable chelal finger with 92–94 teeth; pedipalpal fixed chelal finger with 95–98 teeth.

#### Description.

**Adult male** (holotype and male paratypes) (Figs 13A, 14B).

***Carapace*** (Figs 15A, 16A). Carapace 1.08–1.16 times longer than broad, with a total of 30–32 setae, including 5–6 near anterior margin and six near posterior margin; with six lyrifissures near the anterior eyes, four lyrifissures near posterior margin; epistome small, triangular, with rounded top; with four corneate eyes; the anterior half of the carapace uplifted and protruded to the sides, the front half significantly wider than the back part.

***Chelicera*** (Figs 15B, 16B). Hand with seven setae and two lyrifissures; movable finger with one seta; fixed finger with 13–15 teeth; movable finger with 5–6 teeth; serrula exterior with 40–44 lamellae; serrula interior with 36–38 lamellae; galea developed, divided into three main branches, two main branches consisting of two forks each, and another with three forks (Fig. 15D); rallum consisting of eight blades, all with anteriorly directed spinules, the basal-most blade shortest (Fig. 15C).

***Pedipalps*** (Figs 15G, H, 16C, E). Apex of pedipalpal coxa rounded, with 6–7 long setae. Femur with a tubercle in the median area, a big projection on the subdistal prolateral surface, as well as a hook-shaped process near the base of big projection (Figs 15G, 16E); patella smooth (Figs 15G, 16E); chelal hand with 17–19 small triangular, spinous apophyses at the dorsal side of distal half, each spinous apophysis with a seta at the base; on the posterior side, few small granular processes dispersedly located at the distal of the hand and near the base of fingers, and a few dentate bulges at the basal of the fixed finger; fixed chelal finger slightly curved upward at median to distal part (Figs 15H, 16C). Trochanter 1.52–1.83, femur 3.89–5.70, patella 3.53–4.05, chela with pedicel 4.67–4.98, chela without pedicel 4.50–4.80 times longer than broad, movable finger 1.74–2.02 times longer than hand without pedicel. Fixed chelal finger with eight, movable chelal finger with four trichobothria: *eb* and *esb* situated on the base of hand, grouped very closely with *ib* and *isb*; *est*, *et* and *it* grouped distally; *ist* situated midway between *isb* and *it*; *b* and *sb* situated closer to each other in basal half, *st* and *t* close to each other in distal half of movable finger. Venom apparatus present only in fixed chelal finger, venom duct short. Fixed chelal finger with 97–99 pointed teeth, movable finger with 91–95 teeth, 45–57 rounded teeth at base, and 38–46 pointed ones.

***Abdomen*.** Pleural membrane granulated. Tergites and sternites undivided, tergal chaetotaxy (I–XI): 4–5: 8–9: 9–11: 9–11: 10–11: 9–11: 9–12: 11–13: 11–12: 8–10: 8–10, sternal chaetotaxy (IV–XI): 23–26: 19–20: 15–19: 13–19: 15–16: 12–14: 10–12: 4–5, sternites VI–VIII with 9–13 medial scattered glandular setae, anal cone with two dorsal and two ventral setae. Genital area (Figs 15F, 16D): sternite II with total of 30–35 setae and two lyrifissures; sternite III with 46–56 setae.

***Legs*** (Figs 15I, J, 16F–I). The femur with an inward depression at the distal of the leg I, leg I patella enlarged (Figs 15I, 16F), and fusing (Figs 15I, 16F, H) or semi-fusing (Fig. 16I) of the basitarsus and telotarsus, the dividing line between the basitarsus and telotarsus inconspicuous or slightly visible. Leg IV generally typical, long, and sinewy (Figs 15J, 16G). Leg I: trochanter 1.05–1.16, femur 3.47–5.52, patella 2.53–2.90, tibia 3.45–4.29, basitarsus + telotarsus 6.64–6.80 times longer than deep. Leg IV: trochanter 2.36–2.78, femur + patella 4.08–4.88, tibia 6.80–7.56, basitarsus 4.23–4.85, telotarsus 6.62–8.40 times longer than deep; tibia with three submedial tactile setae (TS = 0.16, 0.61, 0.92), basitarsus with two tactile setae (TS = 0.14, 0.83–0.84), telotarsus with two tactile setae (TS = 0.24–0.30, 0.58–0.60); subterminal tarsal seta bifurcate (Fig. 15E). Arolium not divided, shorter than the slender and simple claws.

**Adult female** (paratype females) (Figs 13B, 14B): mostly same as males, except where noted.

***Carapace*** (Figs 17A, 18A). Smooth and nearly rectangular, 1.02–1.10 times longer than broad, with a total of 27–31 setae, including 6–7 near anterior margin and 6–7 near posterior margin; with two pair lyrifissures near the anterior eyes, two lyrifissures near posterior margin.

***Chelicera*** (Figs 17B, 18B). Fixed finger with 12–13 teeth; movable finger with seven teeth; serrula exterior with 42–45 lamellae; serrula interior with 35–37 lamellae; galea developed, divided into two main branches, one branch five, while the other three (Fig. 17D).

***Pedipalps*** (Figs 17G, H, 18D, E). Apex of pedipalpal coxa with six long setae. Femur with some granular projections; trochanter 1.71–2.02; femur 4.90–5.39; patella 3.39–3.46; chela with pedicel 4.13–4.42; chela without pedicel 3.93–4.29 times longer than broad; movable finger 1.62–1.66 times longer than hand without pedicel. Fixed chelal finger with 95–98 pointed teeth, movable finger with 92–94 teeth, 47–48 rounded teeth at base, and 45–46 pointed ones.

***Abdomen*.** Tergal chaetotaxy (I–XI): 4–5: 6–7: 8–9: 9–10: 10: 9: 9–10: 11: 9–12: 10–11: 7–10, sternal chaetotaxy (IV–XI): 24–26: 20–23: 16–17: 17–18: 15: 14: 12–13: 4–5, sternites VI–VIII with two medial scattered glandular setae; genital area (Figs 17F, 18C): sternite II with total of 19–23 setae and two lyrifissures; sternite III with a row of 35–37 setae and two lyrifissures along posterior margin.

***Legs*** (Figs 17I, J, 18F, G). Leg I: trochanter 1.42–1.52, femur 4.78–5.95, patella 3.45–3.75, tibia 4.50–4.79, basitarsus 3.29–4.20, telotarsus 5.40–5.55 times longer than deep. Leg IV: trochanter 2.48–2.55, femur + patella 4.41–4.58, tibia 7.00–7.83, basitarsus 4.69–4.77, telotarsus 7.67–8.08 times longer than deep; tibia with two submedial tactile setae (TS = 0.20, 0.94), basitarsus with two tactile setae (TS = 0.13–0.15, 0.84–0.87), telotarsus with two tactile setae (TS = 0.23–0.26, 0.54–0.59); subterminal tarsal seta bifurcate (Fig. 17E).

#### Measurements

**(in mm; length/breadth or, for legs, length/depth). Male** (holotype and paratypes). Body length 3.66–3.92. Carapace 1.48–1.55/1.31–1.43. Pedipalpal trochanter 0.64–0.75/0.41–0.43, femur 1.75–1.88/0.33–0.45, patella 1.34–1.50/0.37–0.38, chela with pedicel 3.27–3.30/0.66–0.70, chela without pedicel 3.15–3.17/0.66–0.70, hand without pedicel length 1.07–1.20, movable finger length 2.09–2.16. Leg I: trochanter 0.40–0.44/0.38, femur 1.11–1.18/0.21–0.32, patella 1.09–1.13/0.39–0.43, tibia 0.73–0.76/0.17–0.22, basitarsus + telotarsus 0.93–1.03/0.14–0.15. Leg IV: trochanter 0.59–0.71/0.23–0.26, femur + patella 1.55–1.66/0.34–0.38, tibia 1.36–1.44/0.18–0.20, basitarsus 0.55–0.63/0.12–0.13, telotarsus 0.84–0.90/0.10–0.13.

**Female** (paratypes). Body length 4.73–6.31. Carapace 1.34–1.43/1.30–1.32. Pedipalpal trochanter 0.70–0.85/0.41–0.42, femur 1.94–1.96/0.36–0.40, patella 1.42–1.49/0.41–0.44, chela with pedicel 3.35–3.47/0.76–0.84, chela without pedicel 3.26–3.30/0.76–0.84, hand without pedicel length 1.25–1.32, movable finger length 2.07–2.14. Leg I: trochanter 0.37–0.38/0.25–0.26, femur 0.86–1.13/0.18–0.19, patella 0.69–0.75/0.20, tibia 0.67–0.72/0.14–0.16, basitarsus 0.42–0.46/0.10–0.14, telotarsus 0.54–0.61/0.10–0.11. Leg IV: trochanter 0.77–0.84/0.31–0.33, femur + patella 1.74–1.81/0.33–0.41, tibia 1.41–1.47/0.18–0.21, basitarsus 0.61–0.62/0.13, telotarsus 0.92–0.97/0.12.

#### Distribution.

China (Jiangxi).

#### Remarks.

The dividing line between basitarsus and telotarsus of the male leg I of this new species is usually indistinct in specimens examined, except for one paratype, which has this line slightly visible (Fig. 16I). There is no other distinct difference among these male specimens and, as a result, we consider this difference in the visibility of the dividing lines as intraspecific variation.

The males of *S.guangmingensis*, *S.gibba*, and *S.papillata* have a specialized leg I, but this new species can be separated by having a distal depression on leg I femur. Females of this new species can be distinguished from other *Stenohya* species by the following: carapace 1.02–1.10 times longer than broad (1.15–1.28 in *S.curvata*; 1.13 in *S.hainanensis*; 1.33–1.49 in *S.huangi*; 1.15–1.28 in *S.pengae*; 1.18–1.24 in *S.tengchongensis*), the presence of 27–31 setae on carapace (24 in *S.bicornuta* and *S.hainanensis*; 23 in *S.meiacantha* and *S.tengchongensis*; 39–42 in *S.papillata*); the presence of six long setae on apex of pedipalpal coxa (eight in *S.bicornuta* and *S.papillata*; 10 in *S.spinata*), the pedipalpal patella 3.39–3.46 times longer than broad (2.81–2.86 in *S. arcuatа*; 4.70–5.31 in *S.huangi*; 2.68 in *S.meiacantha*; 3.83–3.93 in *S.pengae*; 3.53–3.62 in *S.spinata*; 2.63–2.67 in *S.tengchongensis*); the presence of 92–94 teeth on pedipalpal movable chelal finger (115–119 in *S. arcuatа*; 68 in *S.bomica*; 46–51 in *S.huangi*; 76 in *S.meiacantha*; 79–87 in *S.gibba*; 45–55 in *S.pengae*; 76–78 in *S.spinata*); and the presence of 95–98 teeth on pedipalpal fixed chelal finger (124–129 in *S. arcuatа*; 105 in *S.bicornuta*; 76 in *S.bomica*; 63–69 in *S.huangi*; 84 in *S.meiacantha*; 66–79 in *S.pengae*) (Zhao and Zhang 2011; Zhao et al. 2011; Hu and Zhang 2012; Yang and Zhang 2013; Guo and Zhang 2016; Guo et al. 2019; Zhan et al. 2023).

## ﻿Discussion

In addition to sexually dimorphic pedipalp, the three new species described here a have uniquely sexual dimorphic leg I; that is, the femur and patella are enlarged or have an inward depression, and the basitarsus and telotarsus are fused or semi-fused in males. In particular, the male of *S.gibba* has a large columnar projection on the basitarsus and telotarsus of leg I, which has not been reported in other *Stenohya* species. According to Zhan et al. (2023) the three potential functions of the sexually dimorphic pedipalp are controlling the female during mating, attracting a female during courtship, or serving as a weapon in male-to-male competition. Given the proximity of the pedipalp and leg I, the specialized leg I may interact with the pedipalp in some manner while conducting any of these three potential functions. The discovery of new species enriches our knowledge of the morphological diversity of *Stenohya* pseudoscorpions. The various sexually dimorphic structures imply that *Stenohya* species may have differing adaptive methods under sexual or natural selection.

### ﻿Updated key to the genus *Stenohya* species from China (modified from Zhan et al. 2023)

**Table:** 

1	Male leg I enlarged	**2**
–	Male leg I not enlarged	**4**
2	Male basitarsus and telotarsus of leg I each with a large columnar projection on the lateral side	***S.gibba* sp. nov.**
–	Male basitarsus and telotarsus of leg I without large projections	**3**
3	Male pedipalpal chelal hand with a papillary projection on the ventral face; femur of leg I straight	***S.papillata* sp. nov.**
–	Male femur of leg I with an inward depression at the distal part	***S.guangmingensis* sp. nov.**
4	Male pedipalpal femur and/or patella with projections on prolateral surfaces	**5**
–	Male pedipalpal femur and patella without prolateral projections	**7**
5	Male pedipalpal femur and patella with strong long peg-like projections on prolateral surfaces	***S.spinata* Zhan, Feng & Zhang, 2023**
–	Male pedipalpal patella normal, femur with tubercles on prolateral face	**6**
6	Chelal hand with 14 tooth-shaped tubercles	***S.dongtianensis* Li & Shi, 2023**
–	Chelal hand with 42 tooth-shaped tubercles	***S.jiahensis* Li & Shi, 2023**
7	Male pedipalpal chelal hand with projection on prolateral surface	**8**
–	Male pedipalpal chelal hand without prolateral projection	**10**
8	Prolateral projection of male chelal hand with 2 hornlike bulges	***S.bicornuta* Guo, Zang & Zhang, 2019**
–	Prolateral projection of male chela hand with pointed projection	**9**
9	Male pedipalpal femur with a depression at the base of prolateral face; movable finger basally curved in ventral view	***S.curvata* Zhao, Zhang & Jia, 2011**
–	Male pedipalpal with straight femur; movable finger straight or slightly procurved	***S.meiacantha* Yang & Zhang, 2013**
10	Male pedipalpal femur strongly procurved	**11**
–	Male pedipalpal femur straight or slightly procurved	**12**
11	Male apex of pedipalpal coxa only with 4 long setae, short acicular seta absent	***S.arcuata* Guo, Zang & Zhang, 2019**
–	Male apex of pedipalpal coxa with 3 long setae and 10–12 short acicular ones	***S.setulos* a Guo & Zhang, 2016**
12	Each of chelal fingers with more than 85 teeth	**13**
–	Each of chelal fingers with less than 85 teeth	**14**
13	Male pedipalpal femur distally thickened, noticeably thicker than the basal section	***S.tengchongensis* Yang & Zhang, 2013**
–	Male pedipalpal femur not distally thickened	***S.hainanensis* Guo & Zhang, 2016**
14	Pedipalpal patella 4.0–6.0 times longer than broad	**15**
–	Pedipalpal patella 2.5–3.0 times longer than broad	***S.bomica* Zhao & Zhang, 2011**
15	Carapace with more than 30 setae	**16**
–	Carapace with less than 30 setae	***S.xiningensis* Zhao, Zhang & Jia, 2011**
16	Movable chelal finger with less than 50 teeth; galea divided into 4 or 5 branches	***S.huangi* Hu & Zhang, 2012**
–	Movable chelal finger with more than 50 teeth; galea divided into 6 branches	***S.pengae* Hu & Zhang, 2012**

## Supplementary Material

XML Treatment for Stenohya gibba

XML Treatment for Stenohya papillata

XML Treatment for Stenohya guangmingensis

## ﻿Additional information

### Author ORCIDs

Jiaqi Zhao https://orcid.org/0009-0001-4397-1697

Xiangbo Guo https://orcid.org/0000-0002-7074-8642

Feng Zhang https://orcid.org/0000-0002-3347-1031

### Data availability

All of the data that support the findings of this study are available in the main text.
